# Colonoscopic screening is associated with reduced Colorectal Cancer incidence and mortality: a systematic review and meta-analysis

**DOI:** 10.7150/jca.46661

**Published:** 2020-08-15

**Authors:** Jiaxin Zhang, Guang Chen, Zhiguo Li, Peng Zhang, Xiaoke Li, Da'nan Gan, Xu Cao, Hongbo Du, Jiaying Zhang, Ludan Zhang, Yong'an Ye

**Affiliations:** 1 Dongzhimen Hospital, Beijing University of Chinese Medicine, Beijing, China.; 2 Institute of Liver Diseases, Beijing University of Chinese Medicine.; 3 Ministry of Education Key Laboratory of Bioinformatics, Tsinghua-Peking Center for Life Sciences, School of Life Sciences, Tsinghua University, Beijing 100084, China.

**Keywords:** colorectal cancer, colonoscopy, incidence and mortality, meta-analysis

## Abstract

It is the great priority to detect colorectal cancer (CRC) as early as possible, finally to reduce the incidence and mortality of CRC. However, although colonoscopy is recommended in many consensuses, yet no one systematic review is conducted to figure out how colonoscopy could change the incidence and mortality. In our study, we conducted a comprehensive meta-analysis to evaluate the association between colonoscopy screening and the incidence or mortality of CRC. PubMed, EMBASE, and PMC database were systematically searched from their inception to June 2020. A total of 13 cohort and 16 case-control studies comprising 4,713,778 individuals were obtained in this review. Our results showed that colonoscopy was associated with a 52%* RR* reduction in incidence of CRC (*RR:* 0.48, 95% CI: 0.46-0.49) and 62% *RR* reduction in mortality of CRC (*RR:* 0.38, 95% CI: 0.36-0.40). Subgroup analysis of different interventions, study design, country, sample size, age or sex showed that the incidence and mortality reduction remained consistent, and colonoscopy screening had the same effect on people below and above 50. Our study indicated that colonoscopy could significantly reduce the incidence and mortality of CRC.

## Introduction

Colorectal cancer (CRC), one of the most common malignancies 1, is the leading cause of cancer death worldwide 2, 3 and the second most common cause of cancer death in the United States 4. However, more than 85% of the CRC are found to be advanced; thus, their 5-year survival rate is poorly 50% 3, even though both surgery, chemotherapy, and targeted therapy are used actively. Hence, it is the great priority to detect CRC as early as possible, finally to reduce the incidence and mortality of CRC. However, the diagnosis rate of early CRC in China is less than 10% 5.

In order to detect CRC earlier, the American Cancer Society recommends screening for colorectal cancer from the age of 45, based on epidemiological data and mathematical models. Both the fecal occult blood test (FOBT), fecal DNA test and colonoscopy are the mainstream detecting methods. Compared with the other two methods, colonoscopy is the gold standard for the diagnosis of CRC, and it could meanwhile provide an opportunity for treatment 6, 7. Although several studies have shown that endoscopy could reduce the incidence and mortality of CRC 8-14, and colonoscopy is strongly recommended to prevent CRC by early detection of cancer in the Asia Pacific Consensus 15, yet the quality of evidence is II-2, and the classification of recommendation is B due to the fact that sample size of the original studies supporting the evidence is relatively small, and the strong large-scale randomized trials are still ongoing 16-18.

Although it is recommended in the consensus, colonoscopy screening programs have not been widely implemented in many European countries 19, 20 and Asia-Pacific regions 15. Even in the United States and Germany where screening programs started in the very early years, the screening rate was only 54% by 2013 21 and 20~30% by 2012 22 respectively. Apart from the high costs and lack of colonoscopy professionals, another reason is that actually no one systematic review is conducted to figure out how colonoscopy could change the incidence and mortality. As a result, neither the doctors nor the patients could consider about the balance between the potential benefits and harms of receiving colonoscopy. Recently many case-control and cohort studies based on larger-scale data have reported that colonoscopy might reduce CRC mortality 23-26 in patients with left-sided colon cancer 8, 27. Therefore, this systematic review and meta-analysis was conducted to evaluate the association between colonoscopy screening and the incidence or mortality of CRC.

## Materials and Methods

This systematic review and meta-analysis was conducted on the basis of the Meta-Analysis of Observational Studies in Epidemiology (i.e., MOOSE) and Preferred Reporting Items for Systematic Reviews and Meta-analysis (PRISMA) statement 28, 29. The protocol has been registered at PROSPERO (CRD42019122795, http://www.crd.york.ac.uk/PROSPERO).

### Search Strategy

A comprehensive electronic literature search was performed on PubMed, EMBASE and PMC databases from inception through June 2020 with the following terms: “colonoscopy or colonoscopy” AND “Colorectal Neoplasm or Colorectal Tumor or Colorectal Carcinoma or Colorectal Cancer or Intestinal Neoplasm or Intestinal Cancer or Gastrointestinal Neoplasm or Gastrointestinal Cancer” AND “relative risk or relative risks or odds ratio or odds ratios or rate ratio or rate ratios or risk ratio or risk ratios or hazard ratio or hazard ratios or ratio” AND “case-control studies or cohort studies or cohort or case-control”. The detailed search strategy was described in **Supplementary Tables 1-3.** What's more, we reviewed the references of identified studies for further study. The authors examined the titles and abstracts independently and in duplicate to identify studies that might be eligible and then reviewed the full text to determine trials that met the eligibility criteria.

### Inclusion Criteria and Exclusion criteria

Observational studies (prospective cohort, retrospective cohort, nested case-control, or case-control studies) were included if they met the following criteria: (1) A general population older than 18 years old who are not diagnosed with CRC. (2) The control group did not receive colonoscopy or other examination methods. (3) CRC incidence or mortality confirmed by pathologic diagnosis, and reported risk estimates, such as hazard ratios (*HRs*), relative risks (*RRs*), or odds ratios (*ORs*) with corresponding 95% confidence intervals (*CIs*) or sufficient data for their estimation. (4) Cohort (prospective or retrospective), or case-control studies. As for exclusion criteria, they were displayed as follows: (1) colonoscopy was assessed only in patients with premalignant conditions, colorectal adenoma, inflammatory bowel disease (IBD), ulcerative colitis (UC) and Crohn disease (CD); (2) Non-English published studies; and (3) protocol, case reports, comments, reviews, expert opinions, conference abstracts, letters, and animal experiments.

### Data Extraction and Quality Assessment

For all included studies, the following information was extracted: first author, publication year, design, period, country, sample size, gender, age, intervention, follow-up duration, comparator, frequency and timing of colonoscopic screening, adjustments or matching factors, numbers of outcomes, and effect estimates. The primary outcome was the incidence or mortality of CRC. The quality of each study was evaluated with the Newcastle-Ottawa Quality Rating Scale (NOS), as one of the most useful scales to evaluate the quality of non-randomized studies (http://www.ohri.ca/programs/clinical_epidemiology/oxford.htm). If any disagreement achieved, a third reviewer would join in and reached a consensus.

### Statistical Analysis

Considering low heterogeneities within and between studies, we used the fixed-effects model 30 to calculate the study-specific *RR* estimates. *RR* was employed as a common measure of the association between colonoscopic screening use and the incidence and mortality of CRC. To simplify the terminology, the effect estimates of ORs from case-control studies were directly regarded as an estimate of relative risk (*RR*). Cochrane's Q statistic (*p*<0.10 suggesting significant heterogeneity) and the *I^2^* statistic (*I^2^* > 75.0% representing substantial heterogeneity, 50.0% ≤ *I^2^* ≤ 75.0% representing moderate heterogeneity and *I^2^* < 50% representing low heterogeneity) were adopted to qualitatively and quantitatively evaluate heterogeneity across studies, respectively 31. Sensitivity analysis was conducted by omitting each study in turn. Using Begg's and Egger's test to quantitatively detect publication bias, and the significance level was *p* ≤ 0.1 32, 33. If publication bias was significant, the robustness of meta-analysis results was verified by the trim and fill method 34. All statistical analyses were performed using Stata version 12.0 (StataCorp, College Station, Texas, USA). The statistical significance level was set at a two-sided *p* < 0.05 unless otherwise specified.

## Results

**Figure 1:**
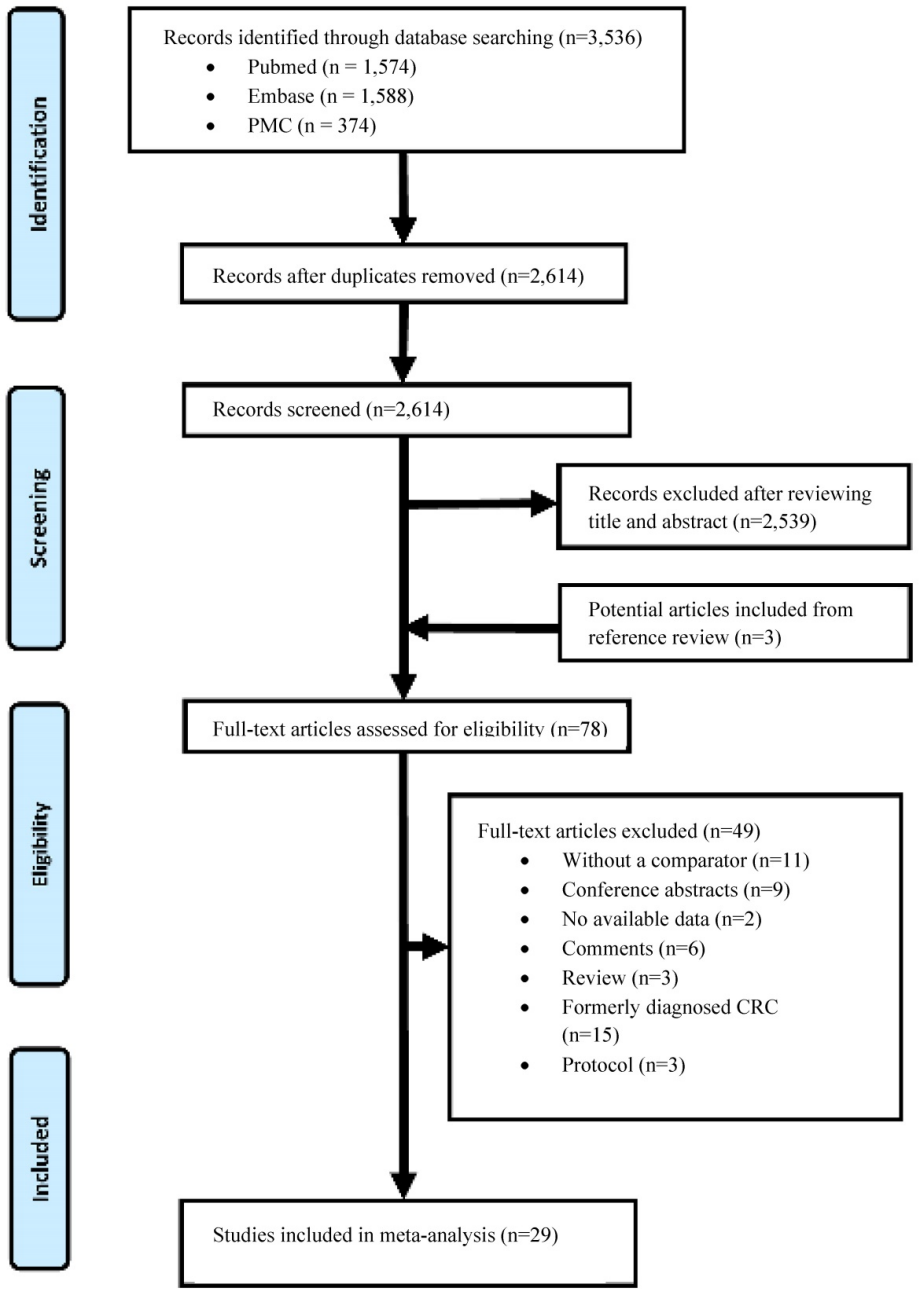
Flow diagram of study selection.

A total of 3,536 studies were included, as is shown in **Figure 1.** After the deletion of duplicate studies, there are 2,614 records were considered potentially relevant. After reviewing the titles and abstracts, a total of 75 articles were considered relevant. Three studies were found to be eligible for inclusion in the manual search process, 78 records left. 49 citations were further excluded after carefully reading the full text. The reasons for exclusion were as follows: without a comparator (n=11), conference abstracts (n=9), no available data (n=2), comments (n=6), review (n=3) and formerly diagnosed CRC (n=15), protocol (n=3). Finally, twenty-nine articles 8, 10, 11, 24, 25, 27, 35-57 were enrolled for meta-analysis.

### Study Characteristics

NOS scores and detailed characteristics of the 29 records are presented in **Supplementary Table 4 & Table 1,** respectively. Among the eligible 29 studies, sixteen were case-control studies 10, 24, 25, 27, 40, 42, 43, 48, 50-57, while the remaining were cohort studies 8, 11, 35-39, 41, 44-47, 49. This meta-analysis included 4,713,778 individuals, three of the studies 35, 38, 47 included more than 1 million individuals, two studies 10, 57 more than 100,000 individuals, ten studies 8, 24, 25, 36, 37, 41, 44-46, 54 included 10,000-100,000 individuals, and fourteen studies 11, 27, 39, 40, 42, 43, 48-53, 55, 56 enrolled less than 10,000 individuals. **Table 2** presented the characteristics of interventions. Among the included studies, there are 19 studies adopted colonoscopic screening, while the remaining study was followed by diagnostic. The regions included in the study were as follows: one from Japan, five from Europe, three from Canada and twenty from the USA. Fourteen and nine studies only reported the incidence or mortality of CRC, respectively, and five reported both.

### Quality of included studies

Quality assessment was shown in **Supplementary Table 4.** Among these 29 eligible studies, the scores of Newcastle-Ottawa quality were ranging from 6 to 8. All studies scored six stars or more. Moreover, most studies were adjusted or matched for the following confounders: age (29/29), sex (29/29) (**Table 1**).

### Incidence reduction of CRC by colonoscopy

**Figure 2:**
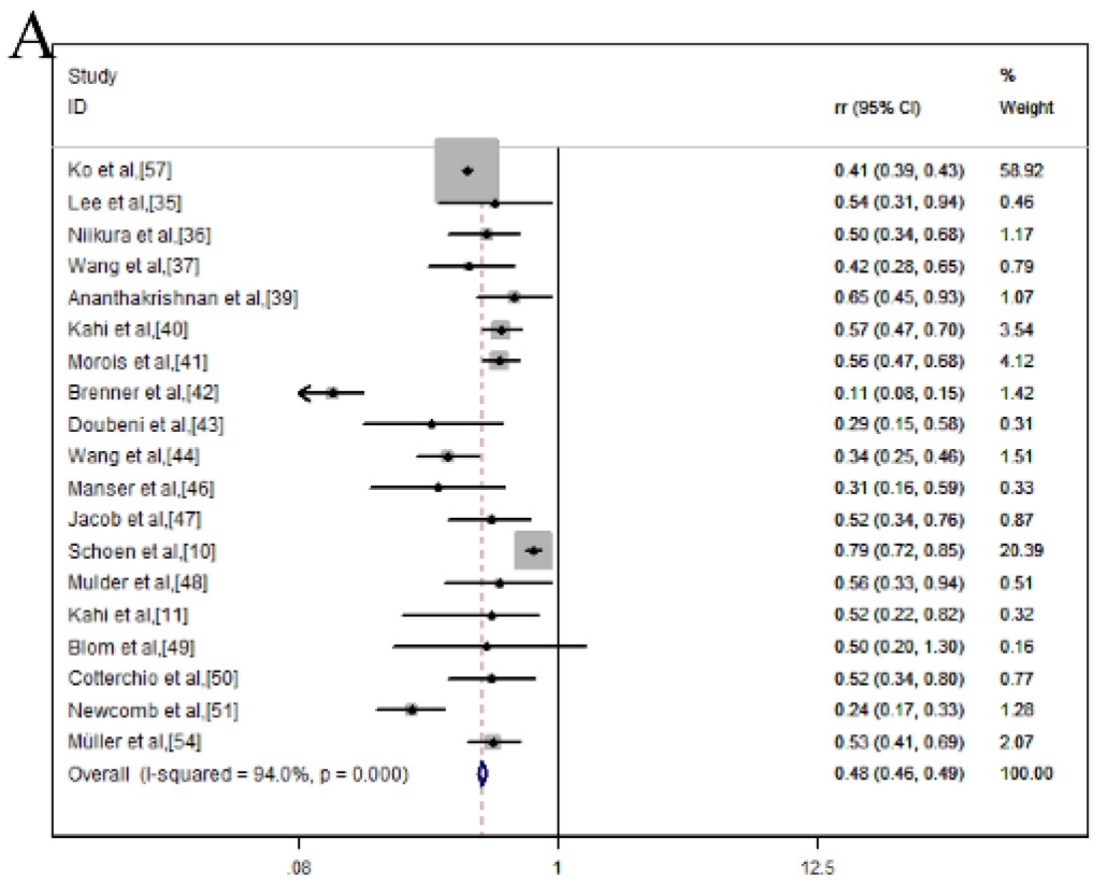

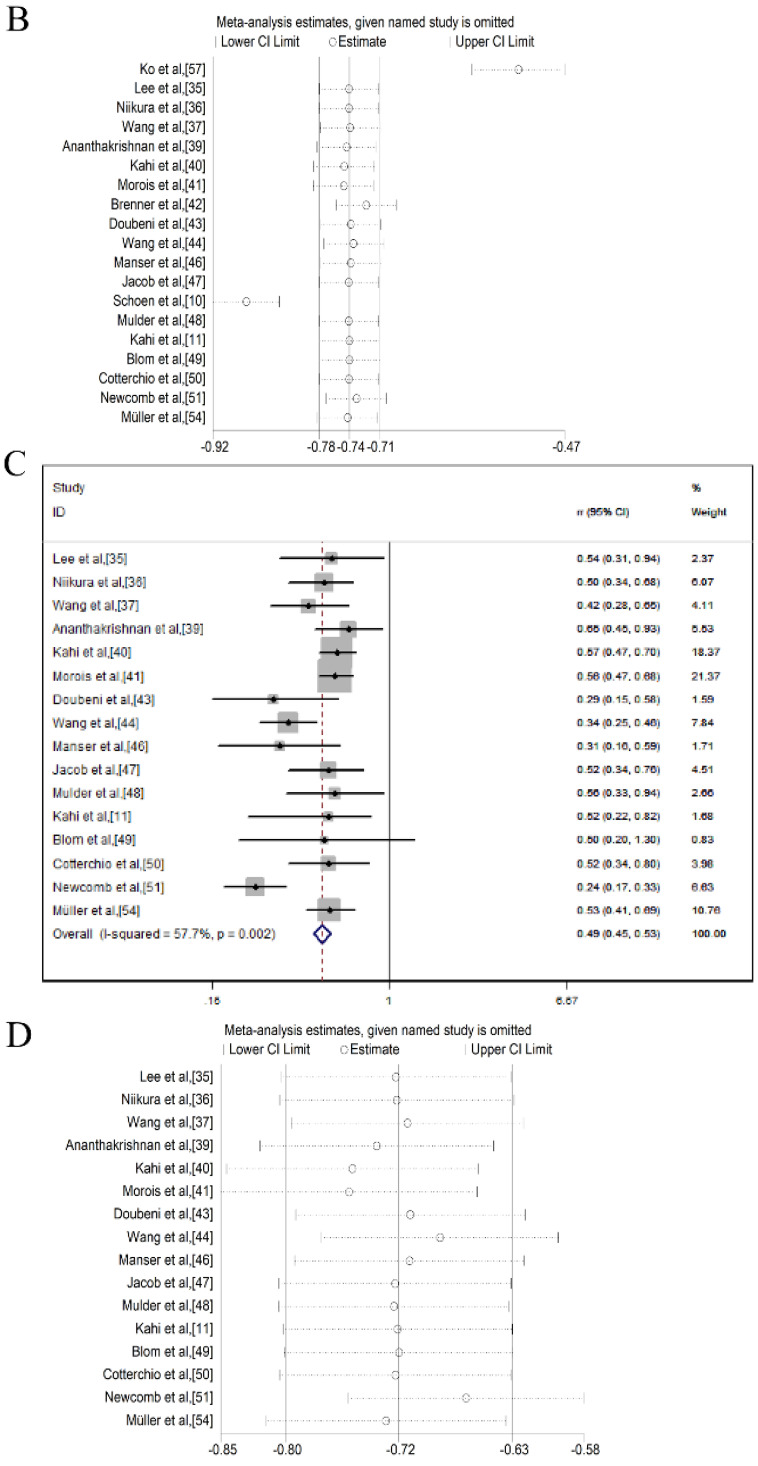
Colonoscopy associated with the incidence reduction of CRC and after excluding one related article; (**A**) Forest plot; (**B**) Sensitivity analysis; (**C**) Forest plot; (**D**) Sensitivity analysis.

As for incidence, a total of 19 studies were calculated the combination of *RR* and 95% *CI* within a fixed-effects model and the values were pooled *RR* = 0.48, 95% *CI* = 0.46-0.49, indicating that colonoscopy can reduce the CRC mortality of 52% *RR* (**Figure 2A**). However, there was high heterogeneity among studies (*I^2^* = 94.0%, *p* = 0.000). To explore the source of heterogeneity, sensitivity analysis was carried out (**Figure 2B**), indicating that the studies of Ko et al, Brenner et al., and Schoen et al., 10, 42, 57 had a great impact on the pooled *RR*. Hence, these three articles were excluded and meanwhile, the incidence rate decreased slightly (*RR*, 0.49; 95% *CI*, 0.45-0.53), as well as the heterogeneity decreased (*I^2^* = 57.7%, *p* = 0.002) (**Figure 2C**). Sensitivity analysis showed that none of these 16 studies could have a great impact on the pooled* RR* (**Figure 2D**).

### Subgroup analysis of CRC incidence reduction

**Figure 3:**
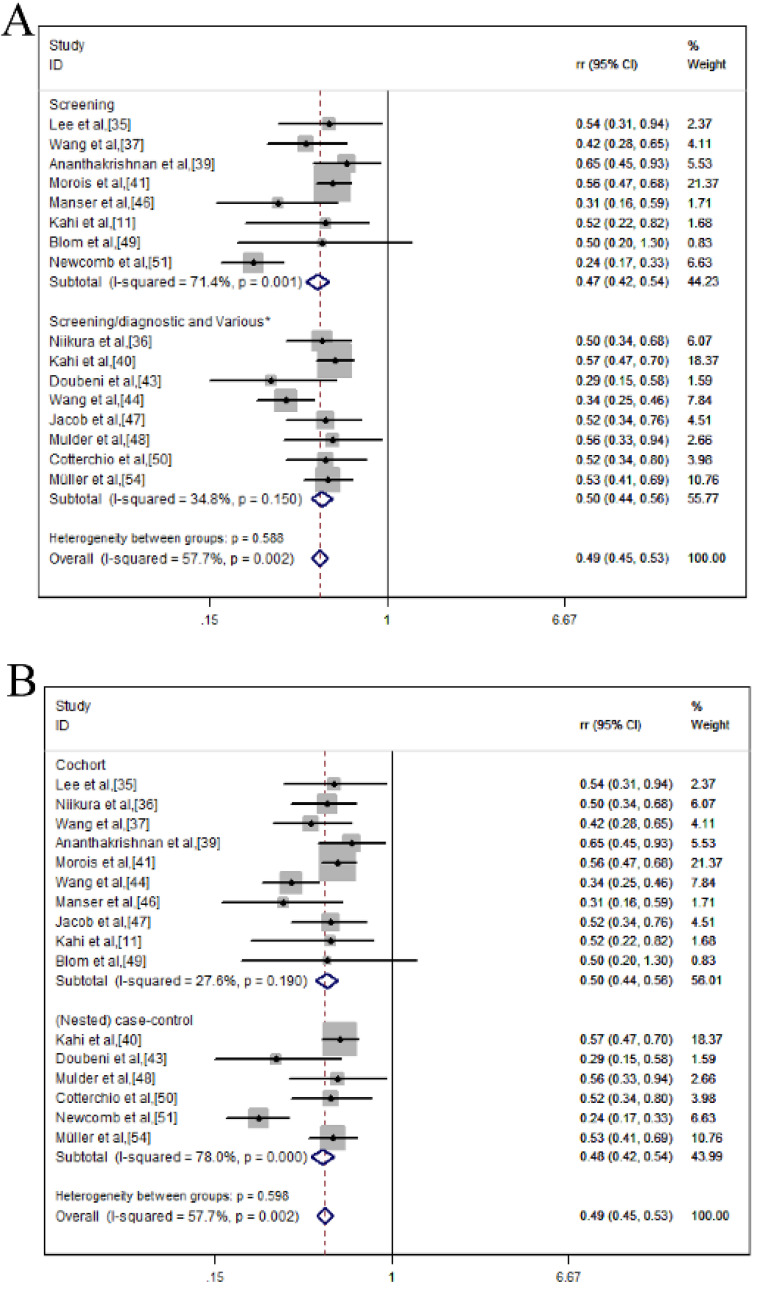

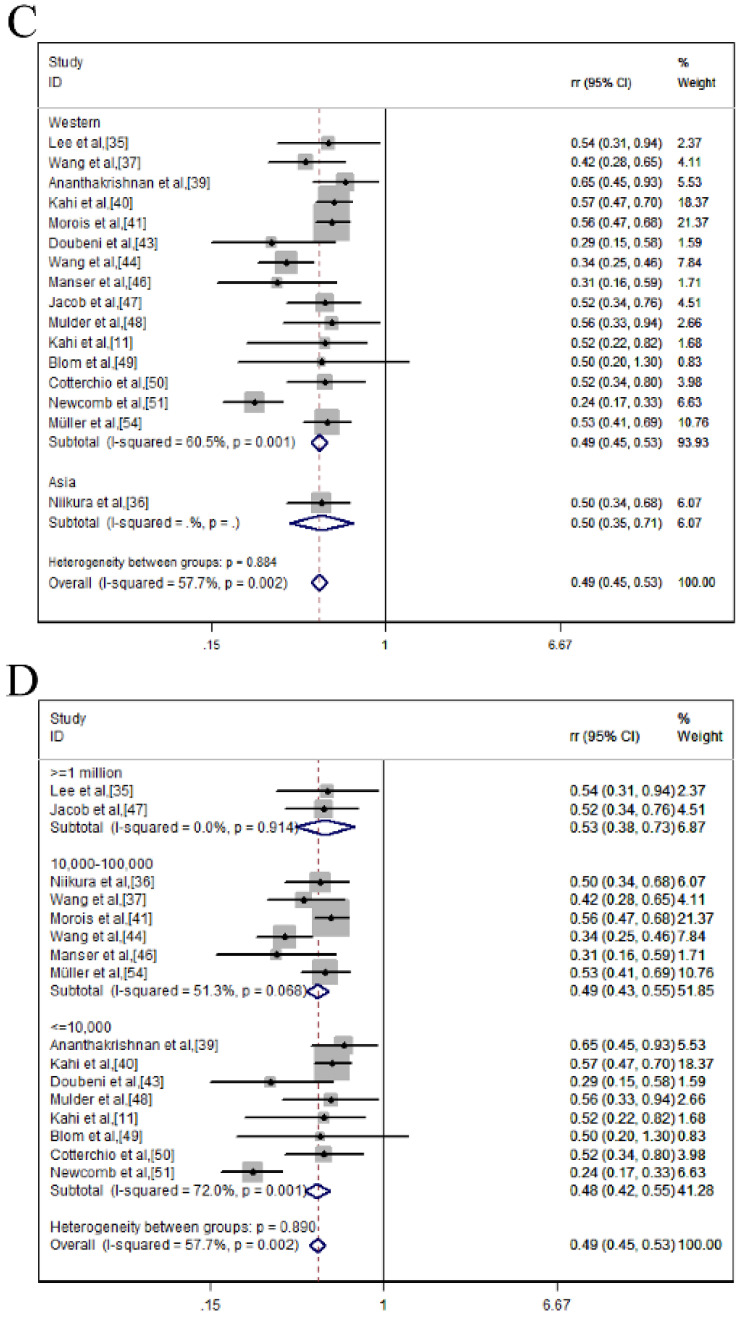

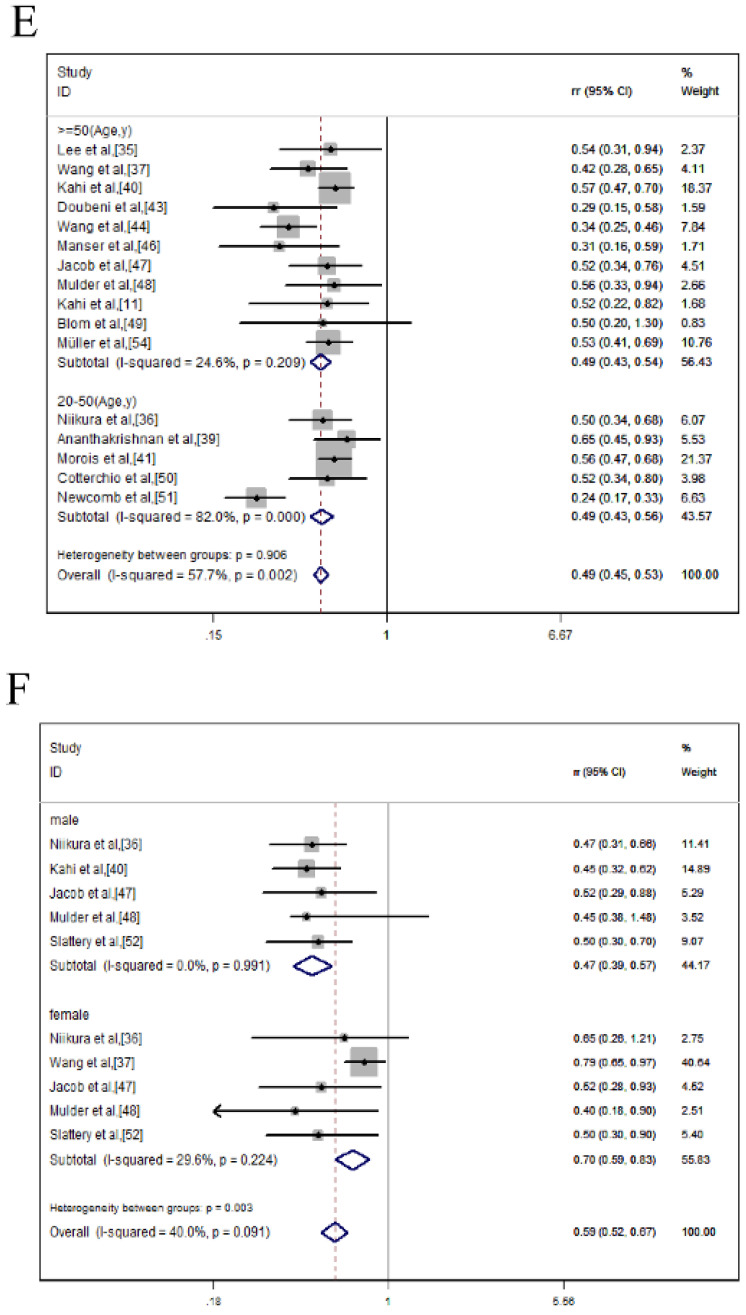
Colonoscopy was associated with a reduced incidence of CRC in a subgroup analysis of forest plots; (**A**) Intervention; (**B**) Study design; (**C**) Country; (**D**) Sample size; (**E**) Age; (**F**) Sex.

As presented in **Table 3 and Figure 3**, we conducted a subgroup analysis of CRC incidence reduction after endoscopic screening based on different interventions, study design, country, sample size, age and sex. We found that colonoscopy could significantly reduce the CRC incidence compared with never-screened (*RR* = 0.475; 95% *CI* = 0.418-0.540; *p* ≤ 0.001; *I^2^* = 71.4%) (**Figure 3A**). In the subgroup analysis of the study design, colonoscopy provided protection in both cohort (*RR*= 0.498; 95 %*CI* = 0.444-0.558;* p* ≤ 0.001; *I^2^* = 27.6%) and case-control studies (*RR* = 0.475; 95% *CI* = 0.418-0.540;* p* ≤ 0.001; *I^2^* = 78.0%) (**Figure 3B**). In the region-based grouping analysis, the incidence of CRC decreased in both western (*RR* = 0.487, 95% *CI* = 0.446-0.532; *p* ≤ 0.001; *I^2^* = 60.5%) and eastern (*RR* = 0.500, 95% *CI* = 0.354-0.707;* p* ≤ 0.001) (**Figure 3C**). Judging from the results of the sample size, colonoscopy can reduce mortality in ≥1 million (*RR* = 0.527, 95% *CI* =0.380-0.730; *p* ≤ 0.001; *I^2^* = 0.0%); 10,000-100,000 (*RR* = 0.486, 95% *CI* = 0.431-0.547; *p* ≤ 0.001; *I^2^* = 51.3%) and less than 10,000 (*RR* = 0.484, 95% *CI* = 0.424-0.553; *p* ≤ 0.001; *I^2^* = 72.0%) (**Figure 3D**). From the age group, colonoscopy screening provided protection in both 20-50 (*RR* = 0.491, 95% *CI* = 0.431-0.558; *p* ≤ 0.001; *I^2^* = 82.0%) and ≥50 (*RR* = 0.485, 95% *CI* = 0.433-0.544; *p* ≤ 0.001; *I^2^* = 24.6%) (**Figure 3E**). Similar results were also shown in sex groups (male: *RR* = 0.473, 95% *CI* = 0.390-0.573; *p* ≤ 0.001; *I^2^* = 0.0%; female: *RR* = 0.702, 95% *CI* = 0.592-0.833; *p* ≤ 0.001; *I^2^* = 29.6%) (**Figure 3F**).

### Mortality reduction of CRC by colonoscopy

**Figure 4:**
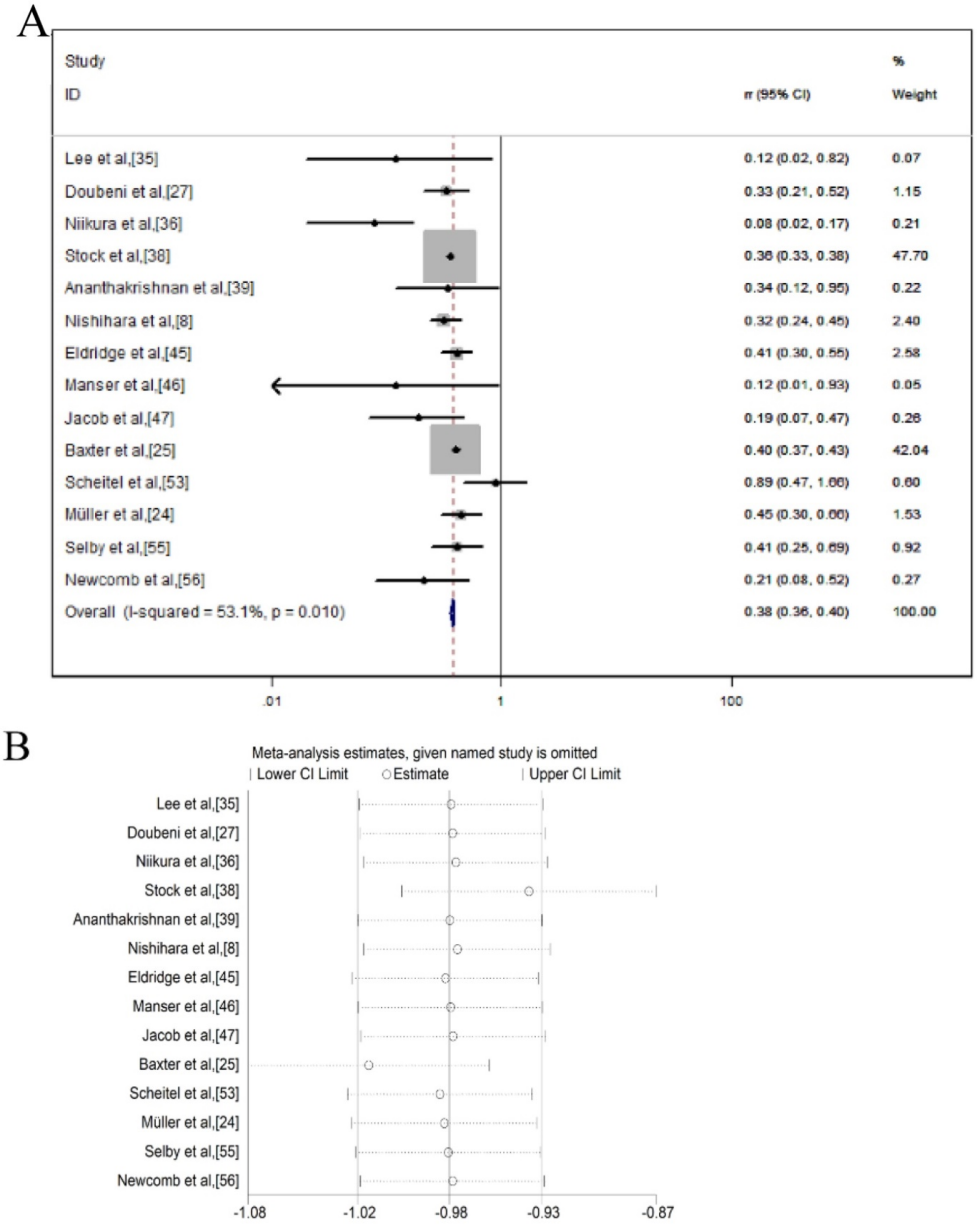
Colonoscopy associated with the mortality reduction of CRC; (**A**) Forest plot; (**B**) Sensitivity analysis.

A total of 14 studies reported a 62% *RR* reduction in CRC morality after the colonoscopic screening within a fixed-effects model. The pooled *RR* was 0.38 (95% *CI* = 0.36-0.40) and the heterogeneity was moderate (*I^2^* = 53.1%, *p* = 0.010) (**Figure 4A**). To assess whether anyone study had a dominant effect on the meta-analysis RR, each study was excluded, and we found no study markedly affected the summary estimate or P-value for heterogeneity among the other summary estimates (**Figure 4B**).

### Subgroup analysis of CRC mortality reduction

**Figure 5:**
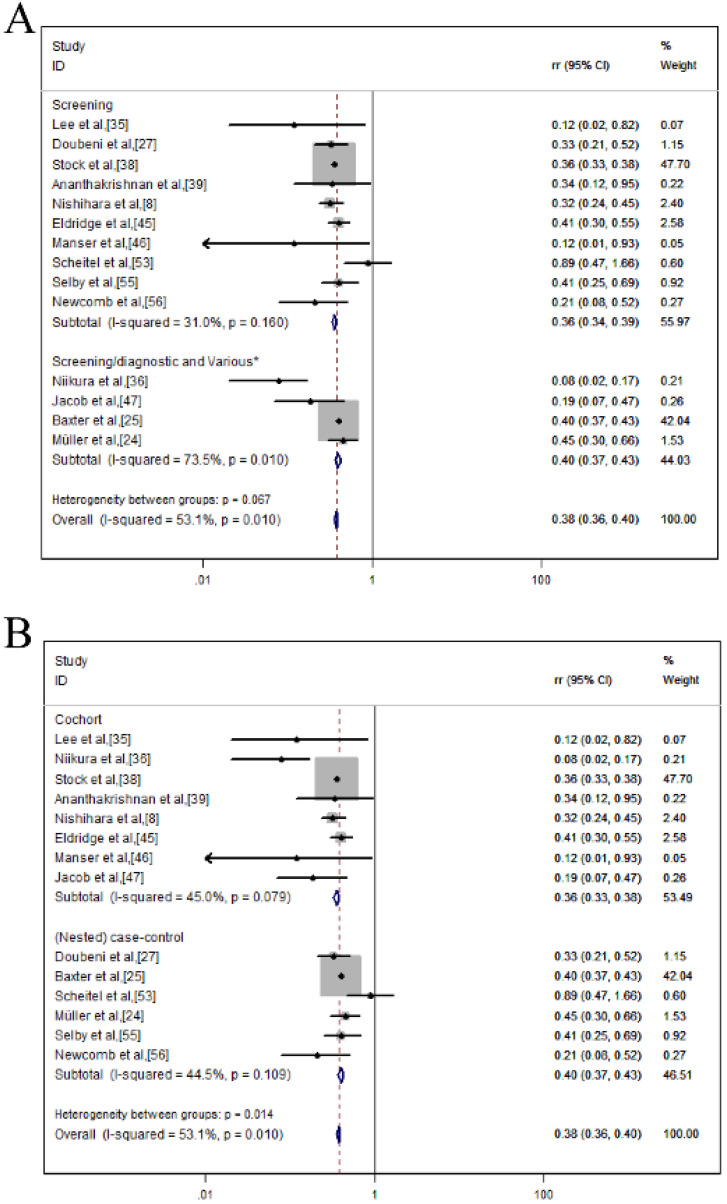

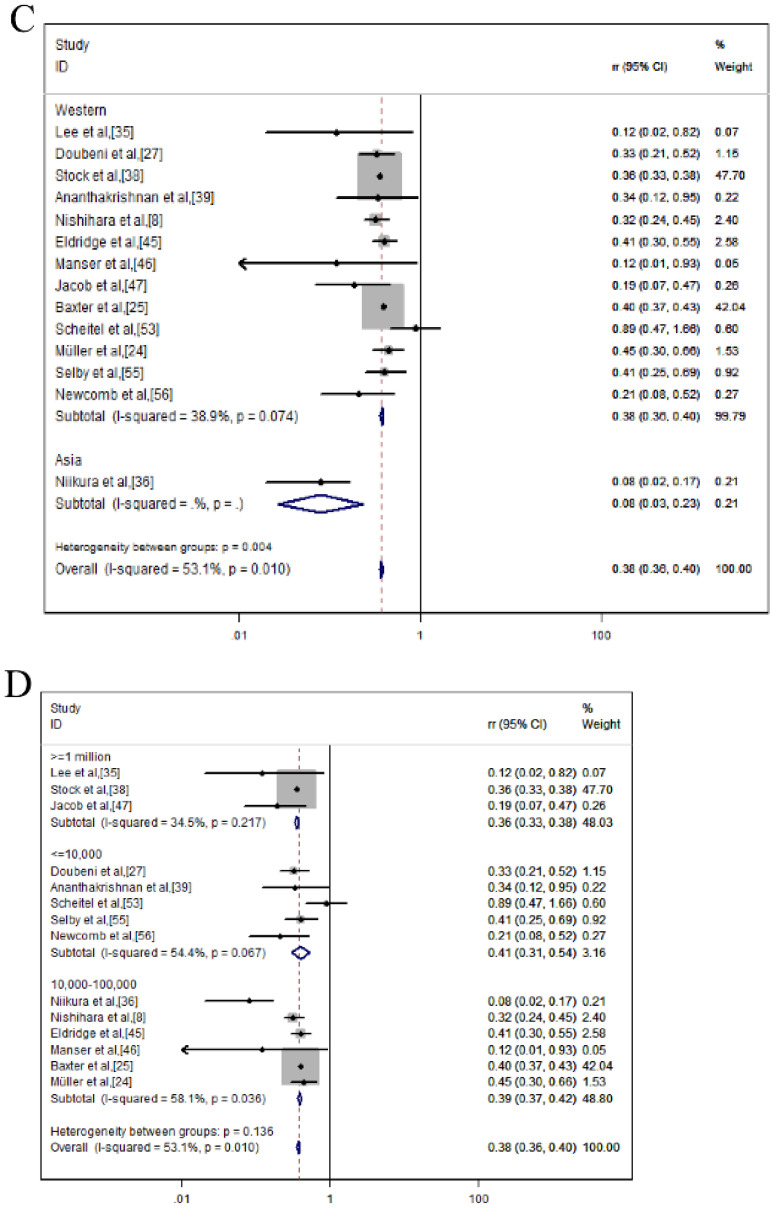

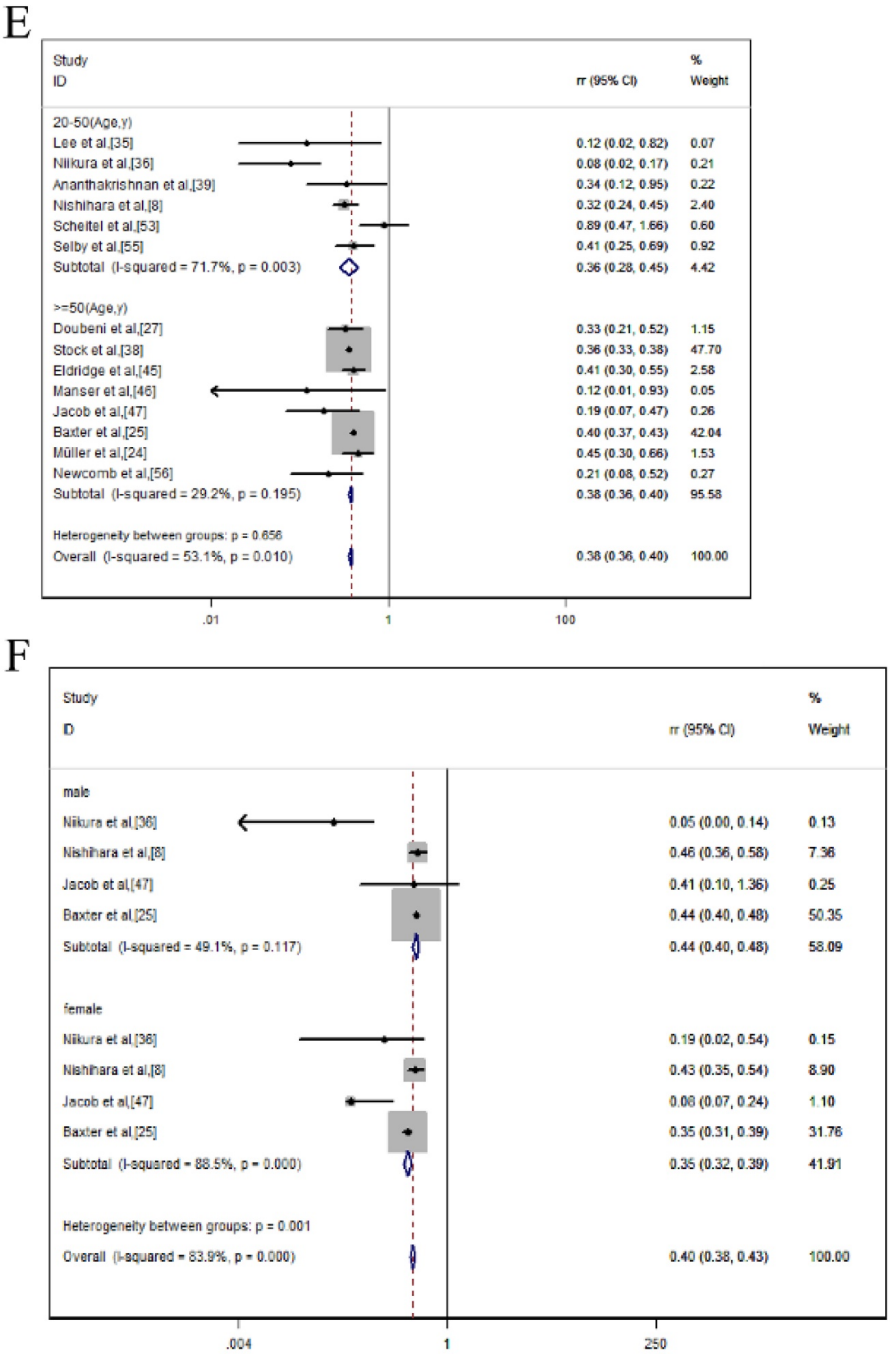
Colonoscopy was associated with a reduced morality of CRC in a subgroup analysis of forest plots; (**A**) Intervention; (**B**) Study design; (**C**) Country; (**D**) Sample size; (**E**) Age; (**F**) Sex.

As displayed in **Table 4 and Figure 5**, we conducted a subgroup analysis of CRC mortality reduction after endoscopic screening based on different interventions, country, sample size, age and sex. We found that colonoscopy screening had a more significant protective effect than never-screened (*RR* = 0.362; 95% *CI* = 0.339-0.386; *p* ≤ 0.001; *I^2^* = 31.0%) (**Figure 5A**). In the subgroup analysis of the study design, colonoscopy provided protection in both cohort (*RR* = 0.356; 95% *CI*= 0.333-0.381;* p* ≤ 0.001; *I^2^*= 45.0%) and case-control studies (*RR* = 0.402; 95% *CI* = 0.375-0.432;* p* ≤ 0.001; *I^2^* = 44.5%) (**Figure 5B**). In terms of country, colonoscopy screening provided protection in both western country (*RR* = 0.378, 95% *CI* = 0.360-0.397; *p* ≤ 0.001; *I^2^* = 38.9%) and eastern country (*RR* = 0.080; 95% *CI* = 0.027-0.233;* p* ≤ 0.001) (**Figure 5C**). Judging from the results of the sample size, colonoscopy can reduce mortality in these three groups (≥1 million: *RR* = 0.358, 95% *CI* = 0.334-0.384, *p* ≤ 0.001, *I^2^* = 34.5%); 10,000-100,000: *RR* = 0.394, 95% *CI* = 0.368-0.423, *p* ≤ 0.001, *I^2^* = 58.1%; ≤10,000: *RR* = 0.409, 95% *CI* = 0.311-0.537, *p* ≤ 0.001; *I^2^* = 54.4%)) (**Figure 5D**). Similar results were also shown in the age (20-50: *RR* = 0.358, 95% *CI* = 0.284-0.451, *p* ≤ 0.001, *I^2^* = 71.7%; ≥50: *RR* = 0.378, 95% *CI* = 0.360-0.397, *p* = 0.001, *I^2^* = 29.2%) (**Figure 5E**) and sex groups (male: *RR* = 0.440, 95% *CI* = 0.404-0.479; *p* ≤ 0.001; *I^2^* = 49.1%; female: *RR* = 0.351, 95% *CI* = 0.318-0.388, *p* ≤ 0.001, *I^2^* = 88.5%) (**Figure 5F**).

### Publication bias

**Figure 6:**
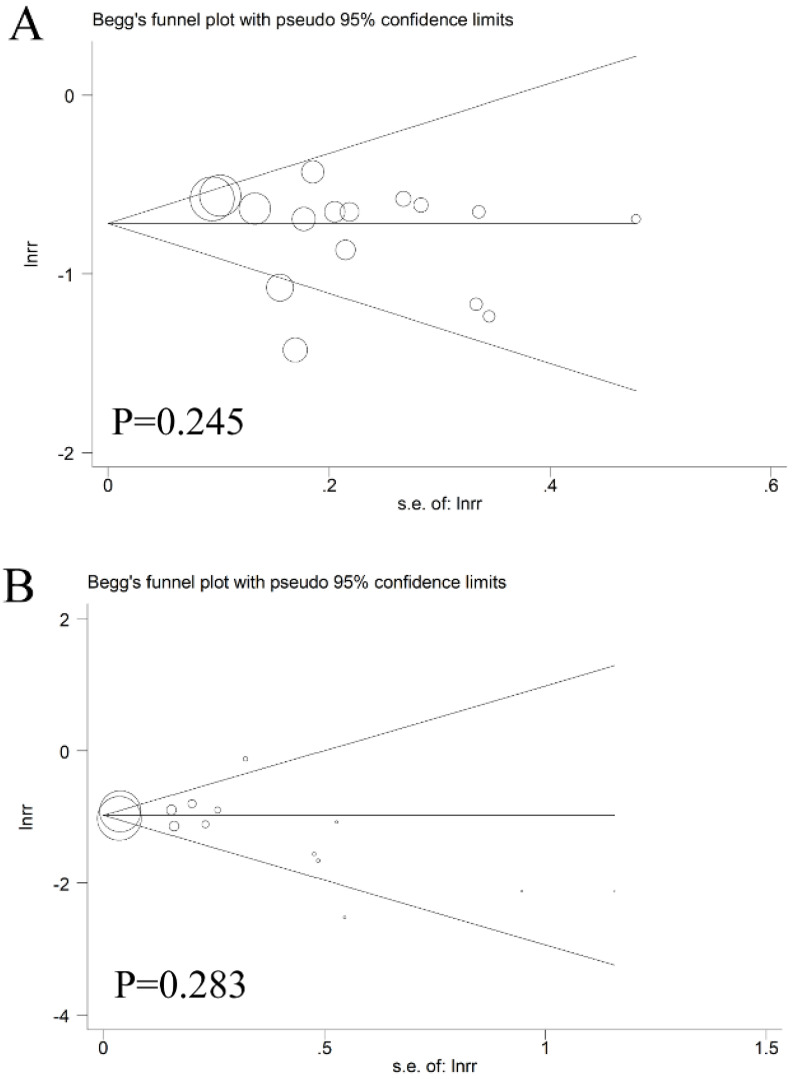
The Begg's funnel plot of the publication bias; (**A**) incidence; (**B**) mortality.

As displayed in **Figure 6**, Begg's test combined with Egger's test was utilized to evaluate the publication bias. In the pooled analysis of CRC incidence or mortality reduction after endoscopic screening, the *p* values of Begg's test and the *p* values of Egger's test were all above 0.05, indicating that there was no obvious bias among these studies.

## Discussion

This meta-analysis set out with the aim of assessing the importance of colonoscopic screening in preventing CRC incidence and related mortality. Of all the 29 studies involving 4,713,778 individuals, our study found a link between colonoscopy and the mortality and incidence of CRC. The outcomes revealed that patients might benefit from 62% *RR* and 52% *RR* reduction in CRC mortality (*RR* = 0.38, 95% *CI* = 0.36-0.40) and incidence (*RR* = 0.48, 95% *CI* = 0.46-0.49) after colonoscopic inspection.

As far as we know, this systematic review and meta-analysis might be one of the leading few studies assessing the value of colonoscopy screening in reducing the risk of CRC among healthy individuals. Moreover, we had observed good results among the associations between colonoscopy screening and the mortality and incidence of CRC. Of all the twenty-nine studies enrolled, only one was from the eastern country. For the remaining 28 studies, we found that colonoscopy could achieve 51% *RR* and 62% *RR* reduction in CRC incidence (*RR* = 0.49, 95% *CI* = 0.45-0.53) and morality (*RR =* 0.38, 95% *CI =* 0.36-0.40) in western countries, which might be a reference for eastern countries. Further prospective studies from China, Japan and Korea are warranted.

Furthermore, the minimum age of regular colonoscopic screening is 50, recommended by developed countries 19, 58-60. It is unclear whether the population under 50 years old could be monitored in the same manner or not. Our study makes up for this gap. The population aged 20-50 years old who underwent colonoscopy was statistically analyzed. We found that colonoscopy could also achieve 64% *RR* and 51% *RR* reduction in CRC mortality (*RR* = 0.36, 95% *CI* = 0.28-0.45; *p*≤0.001) and incidence (*RR =* 0.49*, 95% CI =* 0.43-0.56*, p*≤0.001). We found that colonoscopy screening had a similar protective effect on young people under the age of 50. And we need more data to draw more reliable conclusions.

What's more, direct access colonoscopy service for CRC screening produces a positive financial benefit for patients and local health districts 61. As a clinician, based on our experience, colonoscopy can detect cancer early and have a positive effect on the prognosis of patients, although early colonoscopy is more expensive. However, its cost is lower in the long run, compared with the treatment of advanced cancer. At the same time, from the perspective of social development, it can reduce the direct cost and bring direct economic benefits. For example, colonoscopy early detection, early diagnosis of CRC, patients can receive early treatment, so that he/she can work properly, will increase productivity, bring indirect economic benefits; if the patient does not work, it will increase leisure time. Regardless of the fact that this was not a quantifiable economic benefit, it may be an overall health benefit. These are all pertinent particularly to poorer, developing countries where resources are restricted.

In explaining our findings, attention should be also paid to the following aspects. On the one hand, we did not include randomized controlled trials (RCTs), because it was difficult to conduct RCTs, especially in Japan and Europe, where CRC screening has been introduced into national health programs. What's more, colonoscopy utilization has been on the rise in North America 62-64 and some European countries 65, there are no RCTs results of CRC mortality. The best source of evidence for the reduction in CRC mortality after colonoscopy may be observational studies. On the other hand, the inevitable time deviation may have a certain impact on the assessment of mortality and incidence. Last but not least, some biases are inevitable in observational studies, especially self-selection bias. For example, in the exposure group and the control group of cohort or case-control studies, health-conscious people may receive colonoscopy compared to those who are not, which may overestimate the protective effect of colonoscopy.

Although sensitivity analyses partially explained heterogeneity, the primary source of the heterogeneity is unclear. It could be potentially generated by the inherent relationship between cancer occurrence and the pattern or frequency of colonoscopic inspection. Despite the use of a fixed-effects model in this analysis, it is noteworthy that estimates with high heterogeneity are vulnerable.

## Conclusions

The results indicated that colonoscopy could significantly reduce the incidence and mortality of CRC. After subgroup analysis of different interventions, study design, country, sample size, age or sex, the outcomes remained consistent. Usually, the recommended age by developed countries for regular colonoscopic screening is 50. Based on our results, the population aged 20-50 years old could also benefit from colonoscopic screening. Further researches were required to verify our findings.

## Supplementary Material

Supplementary tables.

## Figures and Tables

**Table 1:** Characteristics and quality of Studies Included in the Meta-analysis

Study	Year	Design	Country	Study period	Sample size	Age,Y	Men (%)	Follow, years	Adjustments or Match	Quality score
Ko et al. 57	2019	CC	USA	2004-2013	133,279 (40,875/92,404)	70-85	44.6	5	1-4,10,15	NOS: 7
Lee et al. 35	2019	RC	USA	1998-2016	1,251,318 (991,945/259,373)	50-75	49	8	1-3,7,10	NOS: 8
Doubeni et al. 27	2018	NCC	USA	2006-2012	5207 (1747/3460)	55-89	49.4	10	1, 2, 13, 15	NOS: 7
Niikura et al. 36	2017	RC	Asia	2001-2015	85,980 (18,816/67,119)	>20	68.51	6	1, 2	NOS: 8
Wang et al. 37	2016	RC	USA	1998-2005	30,138 (5701/24,437)	76-85	65	4	1, 2, 4, 12, 14, 15	NOS: 7
Stock et al. 38	2016	PC	North America	1992-2009	1,509,423 (177,465/1,331,958)	60-80	46.06	8	1, 2, 9, 10	NOS: 7
Ananthakrishnan et al. 39	2015	RC	USA	NR	6823 (2764/4059)	screened 47 (32-61)	45.5	3	1-3	NOS: 7
						Never screened 49 (35-63)				
Kahi et al. 40	2014	CC	USA	1997-2007	2,492 (623/1,869)	81.22±3.89	98.7	5.19	1-3	NOS: 7
Morois et al. 41	2014	PC	Europe	1990-2008	92,048 (37,459/54,589)	screened: 49.9±6.6;	NR	15.4	1,6-8,17	NOS: 7
						control: 48.8±6.6				
Brenner et al. 42	2014	CC	Europe	1993-2010	4,800 (2,516/2,284)	70	59	10	1,2,6-8,15,17	NOS: 7
Doubeni et al. 43	2013	CC	USA	2006-2008	980 (471/509)	55-85	51.4	5	1,2,5,8,9	NOS: 7
Wang et al. 44	2013	RC	USA	1998-2005	53,676 (12,266/41,410)	screened: 73.1±3.8;	39.3	5	1-4,15,17	NOS: 7
						control: 73.3±4.0				
Nishihara et al. 8	2013	PC	USA	1988-2012	88,902 (NA/NA)	Men: 42-77; Women: 32-57	35.7	NR	1,2,6-9	NOS: 7
Eldridge et al. 45	2013	PC	USA	1995-2008	68,531 (22,780/45,751)	50-71	62	11	1-3,5,8,17	NOS: 7
Manser et al. 46	2012	PC	Europe	2000-2007	22,686 (1912/20,774)	50-80	57.8	6	1, 2, 5-8, 16	NOS: 7
Jacob et al. 47	2012	RC	North America	1996-2007	1,089,998 (86,837/1,003,161)	50-74	45.1	7	1,2,4	NOS: 6
Baxter et al. 25	2012	CC	USA	1991-2007	37,099 (9,458/27,641)	screened: 79.9 (70.0-89.9)	42.6	9.4	1-4,15-17	NOS: 7
						control: 79.8 (69.1-90.8)				
Schoen et al. 10	2012	CC	USA	1993-2001	154,890 (77,445/77.445)	55-74	49.5	3	1-3	NOS: 7
Mulder et al. 48	2010	CC	Europe	1996-2005	8384 (594/7790)	69.5±11.9/69.3±11.9	51.7	9	1, 2, 11	NOS: 6
Kahi et al. 11	2009	RC	USA	1989-2007	733 (NA/NA)	61±6.5	59	2	1,2	NOS: 7
Blom et al. 49	2008	PC	Europe	1996-2004	1,986 (NA/NA)	59-61	NR	2	1,2	NOS: 7
Cotterchio et al. 50	2005	CC	North America	1997-2000	2,915 (971/1,944)	20-74	52	2	1,2,5-9,15,17	NOS: 7
Newcomb et al. 51	2003	CC	USA	1998-2002	2,962 (1,668/1,294)	20-74	NR	5	1,2,6-8,17	NOS: 7
Slattery et al. 52	2000	CC	USA	NR	2,893 (1,349/1,544)	30-67	NR	5	1,2,5,7,8	NOS: 8
Scheitel et al. 53	1999	CC	USA	1970-1993	653 (218/435)	45-97	42.2	10	1,2,5,8	NOS: 7
Müller et al. 24	1995	CC	USA	1978-1992	20,889 (4,358/16,531)	Cases (CC): 69.1	97.7	8.3	1-3,8	NOS: 7
						Cases (RC): 68.3				
						Control: 57.0				
Müller et al. 54	1995	CC	USA	1981-1993	32,702 (16,351/16,351)	Cases (CC): 67.2±9.3	97.8	7	1-3	NOS: 7
						Cases (RC): 66.2±9.4				
						Control: 57.0				
Selby et al. 55	1992	CC	USA	1971-1987	1129 (261/868)	40-50	59.4	10	1,2,8	NOS: 6
Newcomb et al. 56	1992	CC	USA	1979-1988	262 (66/196)	50-80	NR	5	1,2,5,8	NOS: 7

Adjusted factors: 1, age; 2, sex; 3, race; 4, income; 5, lifestyle; 6, smoking; 7, body mass index; 8, family history; 9, socioeconomic status; 10, comorbidity; 11, index date; 12, college;13, enrolment duration; 14, Nonwhite; 15, resident city; 16, profession; 17, level of educational. Abbreviations: CC, Case-control; NCC, nested case-control; NR, not reported; PC, prospective cochort; RC, retrospective cohort.

**Table 2:** Results and meta-analyses of observational studies on the effects of colonoscopy on CRC. Values are relative risks (95% confidence intervals) unless stated otherwise

Study	Year	Intervention	Incidence	Mortality
Ko et al. 57	2019	Screening	0.41 (0.39-0.43)	NR
Lee et al. 35	2019	Screening	0.54 (0.31-0.94)	0.12 (0.02-0.82)
Doubeni et al. 27	2018	Screening	NR	0.33 (0.21-0.52)
Niikura et al. 36	2017	Various*	0.50 (0.34-0.68)	0.08 (0.02-0.17)
Wang et al. 37	2016	Screening	0.42 (0.28-0.65)	NR
Stock et al. 38	2016	Screening	NR	0.36 (0.33-0.38)
Ananthakrishnan et al. 39	2015	Screening	0.65 (0.45-0.93)	0.34 (0.12-0.95)
Kahi et al. 40	2014	Screening/diagnostic	0.57 (0.47-0.70)	NR
Morois et al. 41	2014	Screening	0.56 (0.47-0.68)	NR
Brenner et al. 42	2014	Screening	0.11 (0.08-0.15)	NR
Doubeni et al. 43	2013	Various*	0.29 (0.15-0.58)	NR
Wang et al. 44	2013	Screening/diagnostic	0.34 (0.25-0.46)	NR
Nishihara et al. 8	2013	Screening	NR	0.32 (0.24-0.45)
Eldridge et al. 45	2013	Screening	NR	0.41 (0.30-0.55)
Manser et al. 46	2012	Screening	0.31 (0.16-0.59)	0.12 (0.01-0.93)
Jacob et al. 47	2012	Screening/diagnostic	0.52 (0.34-0.76)	0.19 (0.07-0.47)
Baxter et al. 25	2012	Screening/diagnostic	NR	0.40 (0.37-0.43)
Schoen et al. 10	2012	Screening	0.79 (0.72-0.85)	NR
Mulder et al. 48	2010	diagnostic	0.56 (0.33-0.94)	NR
Kahi et al. 11	2009	Screening	0.52 (0.22-0.82)	NR
Blom et al. 49	2008	Screening	0.50 (0.20-1.30)	NR
Cotterchio et al. 50	2005	Various*	0.52 (0.34-0.80)	NR
Newcomb et al. 51	2003	Screening	0.24 (0.17-0.33)	NR
Slattery et al. 52	2000	Screening	NR	NR
Scheitel et al. 53	1999	Screening	NR	0.89 (0.47-1.66)
Müller et al. 24	1995	diagnostic	NR	0.45 (0.30-0.66)
Müller et al. 54	1995	diagnostic	0.53 (0.41-0.69)	NR
Selby et al. 55	1992	Screening	NR	0.41 (0.25-0.69)
Newcomb et al. 56	1992	Screening	NR	0.21 (0.08-0.52)

*Various types analysed separately; NR, not reported.

**Table 3:** Subgroup analysis of CRC incidence reduction after endoscopic screening

Subgroups	No. of studies	Pooled RR (95% CI)	*Z*	*P*	Heterogeneity	
					I2 (%)	Ph
**Intervention**						0.29
Screening 11,35,37,39,41,46,49,51	8	0.475 (0.418-0.540)	5.57	0.000	71.4	
Screening/diagnostic and Various* 36,40,43,44,47,48,50,54	8	0.498 (0.444-0.558)	9.28	0.000	34.8	
Study design						0.28
Cochort 11,35-37,39,41,44,46,47,49	10	0.498 (0.444-0.558)	9.52	0.000	27.6	
(Nested) case-control 40,43,48,50,51,54	6	0.475 (0.418-0.540)	5.28	0.000	78.0	
**Country**						0.02
Western 11,35,37,39-41,43,44,46-51,54	15	0.487 (0.446-0.532)	9.62	0.000	60.5	
Asia 36	1	0.500 (0.354-0.707)	3.92	0.000	NA	
**Sample size**						0.24
≤10,000 11,39,40,43,48-51	8	0.484 (0.424-0.553)	5.23	0.000	72.0	
10,000-100,000 36,37,41,44,46,54	6	0.486 (0.431-0.547)	8.14	0.000	51.3	
≥1 million 35,47	2	0.527 (0.380-0.730)	3.86	0.000	0	
**Age**						0.01
20-50 36,39,41,50,51	5	0.491 (0.431-0.558)	4.46	0.000	82.0	
≥50 11,35,37,40,43,44,46-49,54	11	0.485 (0.433-0.544)	10.23	0.000	24.6	
**Sex**						9.04
Male 36,40,47,48,52	5	0.473 (0.390-0.573)	7.64	0.000	0.0	
Female 36,37,47,48,52	5	0.702 (0.592-0.833)	3.40	0.001	29.6	

NA = not applicable; RR = relative risk; CI = confidence interval.

**Table 4:** Subgroup analysis of CRC mortality reduction after endoscopic screening

Subgroups	No. of studies	Pooled RR (95% CI)	*Z*	*P*	Heterogeneity	
					I2 (%)	Ph
**Intervention**						3.34
Screening 8,27,35,37,39,45,46,53,55,56	10	0.362 (0.339-0.386)	12.11	0.000	31.0	
Screening/diagnostic and Various* 24,25,36,47	4	0.397 (0.369-0.427)	5.09	0.008	73.5	
**Study design**						5.99
Cochort 8,35,36,37,39,45-47	8	0.356 (0.333-0.381)	9.89	0.000	45.0	
(Nested) case-control 24,25,27,53,55,56	6	0.402 (0.375-0.432)	7.87	0.000	44.5	
**Country**						8.08
Western 8,24,25,27,35,37,39,45-47,53,55,56	13	0.378 (0.360-0.397)	18.09	0.000	38.9	
Asia 36	1	0.080 (0.027-0.233)	4.63	0.000	NA	
**Sample size**						3.97
≤10,000 27,39,53,55,56	5	0.409 (0.311-0.537)	4.06	0.000	54.4	
10,000-100,000 8,24,25,36,45,46	6	0.394 (0.368-0.423)	8.94	0.000	58.1	
≥1 million 35,38,47	3	0.358 (0.334-0.384)	4.62	0.000	34.5	
**Age**						0.20
20-50 8,35,36,39,53,55	6	0.358 (0.284-0.451)	4.07	0.000	71.7	
≥50 24,25,27,38,45-47,56	8	0.378 (0.360-0.397)	21.95	0.001	29.2	
**Sex**						11.47
Male 8,25,36,47	4	0.440 (0.404-0.479)	7.00	0.000	49.1	
Female 8,25,36,47	4	0.351 (0.318-0.388)	5.62	0.000	88.5	

NA = not applicable; RR = relative risk; CI = confidence interval.
