# Supplementary material for: Colonoscopic screening is associated with reduced Colorectal Cancer incidence and mortality: a systematic review and meta-analysis
Source: J Cancer. 2020 Aug 15;11(20):5953–70. doi: 10.7150/jca.46661 (PMC7477408; doi:10.7150/jca.46661)
Supplement: Supplementary file 1 — Supplementary tables. [file jcav11p5953s1.pdf]

Supplementary Table 1. Search Strategy Used in PubMed, June, 2020

Number	Search Items	Items Found
1	(((((endoscopy [MeSH Terms]) OR endoscopy) OR colonoscopy [MeSH Terms]) OR colonoscopy) OR sigmoidoscopy [MeSH Terms]) OR sigmoidoscopy	391,093
2	((((((((((((((("Colorectal Neoplasms"[MeSH Terms]) OR "Colorectal Neoplasms"[Title/Abstract]) OR "Colorectal Neoplasm"[Title/Abstract]) OR "Colorectal Tumors"[Title/Abstract]) OR "Colorectal Tumor"[Title/Abstract]) OR "Colorectal Carcinomas"[Title/Abstract]) OR "Colorectal Carcinoma"[Title/Abstract]) OR "Colorectal Cancers"[Title/Abstract]) OR "Colorectal Cancer"[Title/Abstract]) OR "Intestinal Neoplasms"[Title/Abstract]) OR "Intestinal Neoplasm"[Title/Abstract]) OR "Intestinal Cancers"[Title/Abstract]) OR "Intestinal Cancer"[Title/Abstract]) OR "Gastrointestinal Neoplasms"[Title/Abstract]) OR "Gastrointestinal Neoplasm"[Title/Abstract]) OR "Gastrointestinal Cancers"[Title/Abstract]) OR "Gastrointestinal Cancer"[Title/Abstract]	238,275
3	((((((((("relative risk") OR "relative risks") OR "odds ratio") OR "odds ratios") OR "rate ratio") OR "rate ratios") OR "risk ratio") OR "risk ratios") OR "hazard ratio") OR "hazard ratios") OR ratio	1,187,592
4	((("case-control studies"[MeSH Terms]) OR "cohort studies"[MeSH Terms]) OR cohort) OR "case control"	2,460,382
5	Numbers 1–4	1,572

Supplementary Table 2. Search Strategy Used in Embase, June, 2020

Number	Search Items	Items Found
1	'endoscopy'/exp OR 'colonoscopy'/exp OR 'sigmoidoscopy'/exp OR endoscopy OR gastroscopy OR sigmoidoscopy	705,137
2	'colorectal neoplasms'/exp OR 'colorectal neoplasms':ti,ab,kw OR 'colorectal neoplasm':ti,ab,kw OR 'colorectal tumors':ti,ab,kw OR 'colorectal tumor':ti,ab,kw OR 'colorectal carcinomas':ti,ab,kw OR 'colorectal carcinoma':ti,ab,kw OR 'colorectal cancers':ti,ab,kw OR 'colorectal cancer':ti,ab,kw OR 'intestinal neoplasms':ti,ab,kw OR 'intestinal neoplasm':ti,ab,kw OR 'intestinal cancers':ti,ab,kw OR 'intestinal cancer':ti,ab,kw OR 'gastrointestinal neoplasms':ti,ab,kw OR 'gastrointestinal neoplasm':ti,ab,kw OR 'gastrointestinal cancers':ti,ab,kw OR 'gastrointestinal cancer':ti,ab,kw	194,405
3	'risk factor' OR 'relative risks' OR 'odds ratio' OR 'odds ratios' OR 'rate ratio' OR 'rate ratios' OR 'risk ratio' OR 'risk ratios' OR 'hazard ratio' OR 'hazard ratios' OR ratio	2,579,394
4	'cohort analysis'/exp OR 'case control study'/exp OR 'case control' OR cohort	1,226,526
5	Numbers 1–4	1,588

Supplementary Table 3. Search Strategy Used in PMC, June, 2020

Number	Search Items	Items Found
1	(((((endoscopy [MeSH Terms]) OR endoscopy) OR colonoscopy [MeSH Terms]) OR colonoscopy) OR sigmoidoscopy [MeSH Terms]) OR sigmoidoscopy	40,887
2	(((((((((((((("Colorectal Neoplasms"[MeSH Terms]) OR "Colorectal Neoplasms"[Title/Abstract]) OR "Colorectal Neoplasm"[Title/Abstract]) OR "Colorectal Tumors"[Title/Abstract]) OR "Colorectal Tumor"[Title/Abstract]) OR "Colorectal Carcinomas"[Title/Abstract]) OR "Colorectal Carcinoma"[Title/Abstract]) OR "Colorectal Cancers"[Title/Abstract]) OR "Colorectal Cancer"[Title/Abstract]) OR "Intestinal Neoplasms"[Title/Abstract]) OR "Intestinal Neoplasm"[Title/Abstract]) OR "Intestinal Cancers"[Title/Abstract]) OR "Intestinal Cancer"[Title/Abstract]) OR "Gastrointestinal Neoplasms"[Title/Abstract]) OR "Gastrointestinal Neoplasm"[Title/Abstract]) OR "Gastrointestinal Cancers"[Title/Abstract]) OR "Gastrointestinal Cancer"[Title/Abstract]	50,709
3	(((((((((("relative risk") OR "relative risks") OR "odds ratio") OR "odds ratios") OR "rate ratio") OR "rate ratios") OR "risk ratio") OR "risk ratios") OR "hazard ratio") OR "hazard ratios") OR ratio	290,460
4	((("case-control studies"[MeSH Terms]) OR "cohort studies"[MeSH Terms]) OR cohort) OR "case control"	447,923
5	Numbers 1–4	374

Supplementary Table 4: Newcastle-Ottawa Quality Assessments Scale (Newcastle-Ottawa Scale)

Study	Year	Quality Indicators Form Newcastle-Ottawa Scale								Scores
		1	2	3	4	5	6	7	8	
Lee et al,[35]	2019	★	★	★	★	★★	★	★	−	8
Niikura et al,[36]	2017	★	★	★	★	★★	★	★	−	8
Wang et al,[37]	2016	★	★	★	★	★	★	★	−	7
Stock et al,[38]	2016	★	★	★	★	★	★	★	−	7
Ananthakrishnan et al,[39]	2015	★	★	★	★	★	★	★	−	7
Morois et al,[41]	2014	★	★	★	★	★	★	★	−	7
Wang et al,[44]	2013	★	★	★	★	★	★	★	−	7
Nishihara et al,[8]	2013	★	★	★	★	★	★	★	−	7
Eldridge et al,[5]	2013	★	★	★	★	★	★	★	−	7
Manser et al,[46]	2012	★	★	★	★	★	★	★	−	7
Jacob et al,[47]	2012	★	★	★	★	★	★	−	−	6
Kahi et al,[11]	2009	★	★	★	★	★	★	★	−	7
Blom et al,[49]	2008	★	★	★	★	★	★	★	−	7
Ko et al,[57]	2019	☆	☆	☆	☆	☆	☆	☆	−	7
Doubeni et al,[27]	2018	☆	☆	☆	☆	☆	☆	☆	−	7
Kahi et al,[40]	2014	☆	☆	☆	☆	☆	☆	☆	−	7
Brenner et al,[42]	2014	☆	☆	☆	☆	☆☆	☆	−	−	7
Doubeni et al,[43]	2013	☆	☆	☆	☆	☆	☆	☆	−	7
Schoen et al,[25]	2012	☆	☆	☆	☆	☆	☆	☆	−	7
Baxter et al,[10]	2012	☆	☆	☆	☆	☆	☆	☆	−	7
Mulder et al,[48]	2010	☆	☆	☆	☆	☆	☆	−	−	6
Cotterchio et al,[50]	2005	☆	☆	☆	☆	☆	☆	☆	−	7
Newcomb et al,[51]	2003	☆	☆	☆	☆	☆	☆	☆	−	7
Slattery et al,[52]	2000	☆	☆	☆	☆	☆☆	☆	☆	−	8
Scheitel et al,[53]	1999	☆	☆	☆	☆	☆	☆	☆	−	7
Müller et al,[24]	1995	☆	☆	☆	☆	☆	☆	☆	−	7
Müller et al,[54]	1995	☆	☆	☆	☆	☆☆	☆	−	−	7
Selby et al,[55]	1992	☆	☆	☆	☆	☆	☆	−	−	6
Newcomb et al,[56]	1992	☆	☆	☆	☆	☆	☆	☆	−	7

★: Cohort Studies; ☆: Case-Control Studies. The first half of the description is the case-control study entry and the second one is cohort study. 1.Representativeness of the exposed cohort/Definition of Cases; 2.Selection of the non-exposed cohort/Representativeness of Cases; 3.Ascertainment of exposure/Selection of Controls; 4.Outcome of interest not present at start of study/Definition of Controls; 5.Control for important factor or additional factor/Comparability; 6.Assessment of outcome/Assessment of exposure; 7.Follow-up long enough for outcomes to occur/Method of Ascertainment; 8.Adequacy of follow up of cohorts/Non-Response Rate.
